# Anti-obesity effect of extract from fermented *Curcuma longa* L. through regulation of adipogenesis and lipolysis pathway in high-fat diet-induced obese rats

**DOI:** 10.3402/fnr.v60.30428

**Published:** 2016-01-27

**Authors:** Ji Hye Kim, Ok-Kyung Kim, Ho-Geun Yoon, Jeongjin Park, Yanghee You, Kyungmi Kim, Yoo-Hyun Lee, Kyung-Chul Choi, Jeongmin Lee, Woojin Jun

**Affiliations:** 1 Division of Food and Nutritional Science, Chonnam National University, Gwangju, Republic of Korea; 2 Research Institute for Human Ecology, Chonnam National University, Gwangju, Republic of Korea; 3 Department of Biochemistry and Molecular Biology, Brain Korea 21 PLUS Project for Medical Sciences, Yonsei University College of Medicine, Seoul, Republic of Korea; 4 Department of Biofood Analysis, Korea Bio Polytechnic, Nonsan, Chungnam, Republic of Korea; 5 Department of Food and Nutrition, University of Suwon, Suwon, Republic of Korea; 6 Department of Biomedical Sciences, University of Ulsan College of Medicine, Seoul, Republic of Korea; 7 Department of Medical Nutrition, Kyung Hee University, Yongin, Republic of Korea

**Keywords:** *Curcuma longa*, high-fat diet, adipogenesis, lipolysis, anti-obesity

## Abstract

**Background:**

Even though *Curcuma longa* L. possesses various biological activities, it has strong flavor and taste, which decrease consumer palatability and limit industrial applications in food.

**Objective:**

The present study investigates the effects of *C. longa* L. fermented with *Aspergillus oryzae* supplementation in 60% high-fat diet-induced obese rats measured by the activation of adipogenesis and lipolysis.

**Design:**

Rats were divided into four groups (*n=*6 per group) after 1 week of acclimatization: a normal diet group comprised rats fed the AIN76A rodent diet; a high-fat diet-induced obese group with rats fed a 60% high-fat diet; a *Garcinia cambogia* treated group (positive control) with rats fed a 60% high-fat diet with *G. cambogia* 500 g/kg body weight (b.w.)/day; and an fermented *C. longa* L. 50% ethanolic extract treated group (FCE50) with rats fed a 60% high-fat diet with FCE50 500 g/kg b.w./day. Each group received the appropriate vehicle or sample daily by gastric intubation for 12 weeks.

**Results:**

We found that FCE50 administration suppressed b.w. gain and reduced white adipose tissue weight, serum triglyceride (TG), and cholesterol in high-fat diet-induced obese rats. These results can be associated with the suppression of adipocyte differentiation and lipogenesis with a decrease in the mRNA expressions of fatty acid synthase, acetyl-CoA carboxylase, adipocyte protein 2, and lipoprotein lipase induced by FCE50 administration. In addition, FCE50 increased lipolysis and β-oxidation by up-regulating the expression of lipases such as adipose triglyceride lipase, hormone-sensitive lipase, adiponectin, and AMP-activated protein kinase.

**Conclusions:**

These results suggest that FCE50 can be a candidate for the prevention of obesity *via* suppressing adipogenesis and promoting lipolysis.

Obesity has become a serious global public health problem in the past few decades. It is defined as a medical condition with an abnormal accumulation of body fat and is associated with excessive growth and expansion of adipose tissue due to an imbalance between energy intake and expenditure (1). Obesity commonly leads to a risk of developing various life-threatening diseases, including diabetes, cardiovascular disease, hyperlipidemia, hypertension, non-alcoholic fatty liver, and certain cancers (1, 2).

Dietary excess and obesity are associated with the expansion of adipose tissue. The expansion of adipose tissue is induced by adipogenesis and the accumulation of triglycerides in adipocytes (3). Adipogenesis is a differentiation process by which undifferentiated pre-adipocytes are converted to mature adipocytes (3, 4). During adipocyte differentiation, adipogenic transcription factors, such as sterol regulatory element-binding protein-1c (SREBP-1c), peroxisome proliferators-activator receptor-γ (PPAR-γ), and CCAAT/enhancer binding protein-α (C/EBPα), are considered key regulators of adipogenesis (4–6). SREBP-1c stimulates the expression of C/EBPα, PPAR-γ, and several lipogenic enzyme products including fatty acid synthase (FAS) and acetyl-CoA carboxylase (ACC). Activation of lipogenic enzymes induces the conversion of acetyl-CoA into fatty acids and triglycerides as well as tissue uptake of plasma triglycerides (4, 5). In addition, mature adipocytes secrete lipoprotein lipase (LPL), which plays a central role in controlling lipid accumulation by fatty acid uptake into adipose tissue from circulating lipoproteins (7).

Conversely, lipolysis is the process of triglyceride breakdown, which makes it one of the key processes for achieving weight loss (8). Adipose triglyceride lipase (ATGL) is the rate-limiting enzyme for lipolysis in adipocytes. Another important lipase for catalyzing the process of lipolysis is hormone-sensitive lipase (HSL), which is implicated in hormonal regulation by insulin (9). ATGL initiates lipolysis by specifically removing free fatty acids to produce a diacylglycerol, which is then hydrolyzed by HSL (9). In addition, the phosphorylation of AMP-activated protein kinase (AMPK) activates the β-oxidation of fatty acids and inhibits lipid synthesis pathways, such as FA synthesis, through the suppression of SREBP-1c (9–11).

These lipid metabolisms demonstrate that the inhibition of adipogenesis can prevent obesity and that the activation of lipolysis can cure obesity. Accordingly, there is increasing evidence that some natural plants can induce the inhibition of adipogenesis and lipogenesis or the activation of lipolysis (12). One such plant is the root of *Curcuma longa* L., which is widely cultivated mainly in tropical regions of Asia and Africa. It possesses various biological activities such as anti-obesity, anti-atherosclerosis, anti-diabetes, anti-mutagenesis, anti-cancer, and anti-oxidation effects (13). These effects might be related to its well-known biologically active compounds, curcuminoids, which include curcumin, demethoxycurcumin, and bisdemethoxycurcumin (14). Even though *C. longa* L. has good medicinal efficacy, it has strong flavor and taste, which decrease consumer palatability and limit the industrial applications in food. Consequently, it is necessary to improve its characterization. *C. longa* L. fermented by *Aspergillus oryzae* has various properties including reduced bitterness and harsh taste that increase consumer acceptance (15). In the current study, we investigated the effects of *C. longa* L. 50% ethanolic extract supplementation in 60% high-fat diet-induced obese rats measured by the activation of adipogenesis and lipolysis.

## Methods

### Preparation of 50% ethanol extract from fermented *C. longa L.*

Raw *C. longa* L. was harvested from Jindo Jeollanam-do, Korea, and prepared as a 200-mesh powder by grinding and sieving. The powder was fermented with 2% *A. oryzae*. After fermentation for 40 h at the temperature of 25±2°C and humidity of 50±5%, the product was sterilized at 121°C for 15 min and dried at 60±5°C. Finally, the dried sample was prepared as a 200-mesh powder by grinding and sieving. The powder (1 kg) was refluxed with 20 volumes of 50% ethanol at 250°C for 5 h. The extraction solution obtained after filtering was concentrated and lyophilized, yielding 50% ethanol extract (FCE50, 19%). It was stored at −20°C until needed.

### Animals and diets

Five-week-old male Sprague–Dawley (SD) rats with a body weight (b.w.) of 225±2.5 g were purchased from Orient Bio (Seongnam, Korea) and housed in cages under automatically controlled air conditions of temperature (22±2°C), humidity (about 50%), and lighting (12:12-h light/dark cycle). The rats in the normal diet control group were fed a commercial pelleted chow (AIN-76A rodent purified diet, Orient Bio) and water *ad libitum*. For the high-fat diet-induced obese group, rats were fed a 60% high-fat diet (60% high-fat rodent purified diet, Orient Bio). The Institutional Animal Care and Use Committee of Chonnam National University approved the protocol (CNU IACUC-YB-2015-35) for the animal study, and the animals were cared for in accordance with the ‘Guidelines for Animal Experiments’ established by the university.

Rats were divided into four groups after 1 week of acclimatization: a normal diet group (ND, *n=*6) comprised rats fed the AIN76A rodent diet; a high-fat diet-induced obese group (HFD, *n*=6) comprised rats fed a 60% high-fat diet; a *Garcinia cambogia* treated group (positive control) (GC, *n*=6) comprised rats fed a 60% high-fat diet with *G. cambogia* 500 g/kg b.w./day; and a FCE50 treated group (FCE50, *n*=6) comprised rats fed a 60% high-fat diet with FCE50 500 g/kg b.w./day. *G. cambogia* was used for reference. It has been found to contain large amounts of hydroxycitric acid, which is widely used as an anti-obesity herbal supplement (16). Each group received the appropriate vehicle or sample daily by gastric intubation for 12 weeks. Body weight was measured at the beginning of the experiment and at 1-week intervals for 12 weeks. The amount of food consumption by each group was recorded two times per week for 12 weeks. At the end of the experiment, the mice were sacrificed to collect serum and tissues.

### Measurement of serum triglyceride and cholesterols

Serum was separated from blood samples by centrifugation at 3,000*g* for 15 min. The concentrations of serum triglyceride (TG), total cholesterol, low-density lipoprotein (LDL)/very-low-density lipoprotein (VLDL) cholesterol, and high-density lipoprotein (HDL) cholesterol were evaluated spectrophotometrically, using commercially available diagnostic kits supplied by Asan Pharmaceutical (Seoul, South Korea).

### Protein extraction and western blot analysis

Quantitation of AMPK phosphorylation proteins was carried out by western blot analysis. The white adipose tissues of each rat were homogenized in a CelLytic™ MT lysis reagent (Sigma–Aldrich, Sigma, St. Louis, MO, USA) and centrifuged at 12,000*g* for 20 min at 4°C. The protein content of the clear lysates was estimated with the Bradford method using the Protein assay reagent (Bio-Rad Laboratories, Hercules, CA, USA). Forty micrograms of protein were dissolved in NuPAGE^®^ LDS sample buffer 4× (Life Technologies, Gaithersburg, MD, USA). Membranes were incubated for 1 h in a blocking solution containing 5% non-fat milk in Tris-buffered saline and then incubated for 12 h at 4°C with anti-β-actin (1:1,000, Abcam, Cambridge, England) and anti-phospho-AMPK (1:1,000, Abcam). After incubation with the primary antibody, membranes were incubated with a secondary antibody (anti-rabbit IgG HRP-linked antibody, 1:5,000, Cell Signaling, Beverly, MA, USA) for 1 h at room temperature. Proteins were developed using a detection system (Molecular Imager ChemiDoc™XRS+, Bio-Rad Laboratories) and visualized with Image Lab™ Software (Bio-Rad Laboratories).

### Real-time PCR

Total RNA was isolated from epididymal white adipose tissue by Trizol reagent extraction according to the manufacturer's instructions (MRC, Montgomery, OH, USA). Complementary DNA was synthesized from 1 µL purified total RNA in 20 µL of reaction buffer using the iScript™ cDNA Synthesis Kit (Bio-Rad Laboratories). Real-time PCR (Applied Biosystems, Foster City, CA, USA) was performed on triplicate samples using 1 µL cDNA with the SYBR Green PCR Master Mix (iQ SYBR Green Supermix, Bio-Rad Laboratories). The cDNA was amplified for 40 cycles of denaturation (95°C for 30 sec), annealing (57°C for 40 sec) and extension (72°C for 40 sec). The sequences of the primers used in the study are as follows: ACC (XM_132282), F: 5′-TCC CCA AGT TCT TCA CGT TCA-3′, R: 5′-CAG GCT CCA AGT GGC GAT AA-3′; adiponectin (NM_144744), F: 5′-AAC CCC TGG CAG GAA AGG-3′, R: 5′-TGA ACG CTG AGC GAT ACA CAT-3′; aP2 (M84651), F: 5′-TCT CTG GCA GGC ATC TGA CA-3′, R: 5′-GGC AGC AGA CTG TGA TCA ACT T-3′; ATGL (AY894805), F: 5′-CAT TCT CAG GCG AGA GTG ACA T-3′, R: 5′-GAC GCG AAG CTC GTG GAT-3′; C/EBPα (NM_012524), F: 5′-CTG CGA GCA CGA GAC GTC TA-3′, R: 5′-GCC AGG AAC TCG TCG TTG AA-3′; CPT1 (NM_013495), F: 5′-GTG ACT GGT GGG AGG AAT AC-3′, R: 5′-GAG CAT CTC CAT GGC GTA G-3′; PPAR-γ (AB011365), F: 5′-TGA CTT GGC CAT ATT TAT AGC TGT CA-3′, R: 5′-CGA TGG GCT TCA CGT TCA G-3′; FAS (X62888), F: 5′-GAC CCT GAC TCC AAG TTA TTC GA-3′, R: 5′-CGT CAA GCG GGA GAC AGA CT-3′; HSL (BC021642), F: 5′-CAC TAG TCC CTC CCC CAG TTT-3′, R: 5′-AGC TGG CAC AGC AGG TCT GT-3′; LPL (M60838), F: 5′-CAA GAT TCA CTT TTC TGG GAC TGA-3′, R: 5′-GCC ACT GTG CCG TAC AGA GA-3′; GAPDH (NM_017008), F: 5′-TGG CCT CCA AGG AGT AAG AAA C-3′, R: 5′-GGC CTC TCT CTT GCT CTC AGT ATC-3′. The real-time PCR results were visualized and the relative quantitation was calculated using the 7500 System SDS software version 1.3.1 (Applied Biosystems, Foster City, CA, USA).

### Statistical analysis

All data are presented as mean±SD. The data were statistically evaluated using Duncan's multiple range tests after one-way ANOVA using SPSS statistical procedures (SPSS PASW Statistic 20.0, SPSS, Inc., Chicago, IL, USA). Statistical significant differences were considered at the *p*<0.05 level.

## Results

### Effect of FCE50 on b.w. gain and white adipose tissue weight in high-fat diet-induced obese rats

Table 1 shows the effects of FCE50 on the b.w. gain and white adipose tissue weight of the experimental rats. The b.w. gain and food efficiency rate (FER) increased significantly in the HFD group compared to the ND group. FCE50 administration for 12 weeks significantly decreased b.w. gain and FER compared to the HFD group. The weights of epididymal and perirenal white adipose tissues were significantly higher in the HFD group compared to the ND group. The epididymal and perirenal white adipose tissue weights of the FCE50 group were significantly lower than those of the HFD group (*p*<0.05).

**Table 1:** Effect of 50% ethanol extract from fermented *Curcuma longa* L. (FCE50) on body weight gain and white adipose tissue weight in high-fat diet-induced obese rats

			High-fat diet-induced obese		
			
		ND	HFD	GC	FCE50
Initial body weight (g)		223.8±7.2	225.3±4.5	225.7±6.1	225.2±3.4
Final body weight (g)		500±13.1^b^	578.9±10.7^a^	500.1±17.1^b^	487.1±24^b^
Body weight gain (g)		276.3±19.7^b^	353.6±14.9^a^	274.3±23.2^a^	261.9±27.4^a^
FER*		15.8±1.1^c^	25.4±1.1^a^	20.8±1.8^b^	20.0±2.1^b^
White adipose tissue weight (g)	Epididymal	9.4±0.5^b^	16.6±0.9^a^	9.6±1.3^b^	11.0±1^b^
	Perirenal	9.5±0.5^c^	18.5±1.1^a^	11.0±2.1^b^	13.1±1.4^b^

* FER (food efficiency rate)=weight gain (g)/total food consumption (g)×100.

The normal diet group (ND) comprised rats fed the AIN76 diet; the high-fat diet-induced obese group (HFD) comprised rats fed a 60% high-fat diet; the *G. cambogia* treated group (positive control) (GC) comprised rats fed a 60% high-fat diet with *G. cambogia* 500 g/kg b.w./day; the FCE50 treated group comprised rats fed a 60% high-fat diet with FCE50 500 g/kg b.w./day. All data are expressed as mean±standard deviation (*n*=6). Different letters show a significant difference at *p*<0.05 as determined by Duncan's multiple range test.

### Effect of FCE50 on levels of serum TG and cholesterol in high-fat diet-induced obese rats

In the HFD group, the levels of serum TG, total cholesterol, and LDL/VLDL cholesterol increased significantly, whereas the levels of serum HDL cholesterol decreased significantly compared with those of the ND group. The GC and FCE50 groups exhibited a significant decrease in the levels of serum TG, total cholesterol, and LDL/VLDL and an increase in the level of serum HDL cholesterol compared with the HFD group. In the HFD group, the atherogenic index (AI) increased significantly whereas the HDL cholesterol/total cholesterol ratio (HTR) decreased significantly compared with the ND group. However, there were significant decreases in the AI and significant increases in the HTR of the GC and FCE50 groups compared with the HFD group (*p*<0.05; Table 2).

**Table 2:** Effect of 50% ethanol extract from fermented *Curcuma longa* L. (FCE50) on lipid profiles, AI and HTR in high-fat diet-induced obese rats

		High-fat diet-induced obese		
		
	ND	HFD	GC	FCE50
Triglyceride (mg/mL)	70.11±3.77^b^	83.22±4.23^a^	48.50±7.34^c^	46.84±8.62^c^
Total cholesterol (mg/mL)	67.22±7.18^b^	90.16±10.24^a^	70.70±10.14^b^	73.55±6.66^b^
LDL/VLDL cholesterol (mg/mL)	48.00±5.65^b^	67.09±6.80^a^	46.35±14.34^b^	51.76±3.12^b^
HDL cholesterol (mg/mL)	31.61±3.16^a^	23.29±4.32^b^	33.03±5.58^a^	37.34±6.64^a^
AI*	1.15±0.37^b^	3.02±1.09^a^	1.19±0.67^b^	1.01±0.35^b^
HTR (%)†	47.57±7.74^a^	26.29±6.69^b^	48.47±12.31^a^	50.91±9.19^a^

* AI (Atherogenic index)=(Total cholesterol – HDL cholesterol)/HDL cholesterol.

† HTR (%)=HDL cholesterol/total cholesterol×100.

The normal diet group (ND) comprised rats fed the AIN76 diet; the high-fat diet-induced obese group (HFD) comprised rats fed a 60% high-fat diet; the *G. cambogia* treated group (positive control) (GC) comprised rats fed a 60% high-fat diet with *G. cambogia* 500 g/kg b.w./day; the FCE50 treated group comprised rats fed a 60% high-fat diet with FCE50 500 g/kg b.w./day. All data are expressed as mean±standard deviation (*n=*6). Different letters show a significant difference at *p*<0.05 as determined by Duncan's multiple range test.

### Effect of FCE50 on adipocyte differentiation in high-fat diet-induced obese rats

**Fig. 1:**
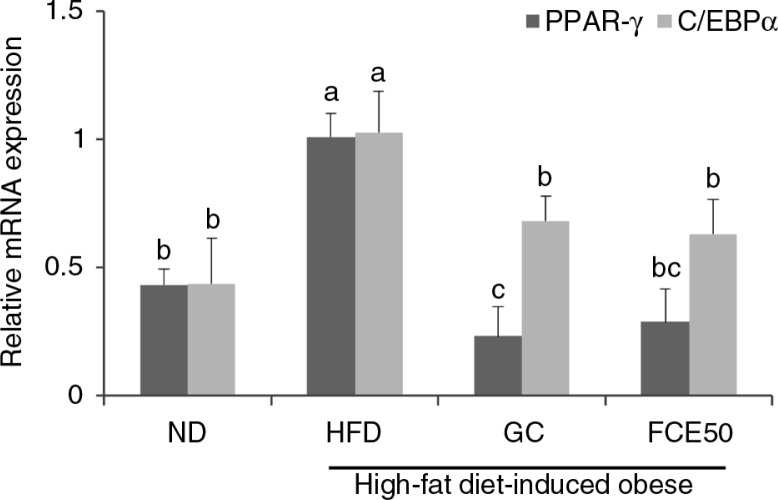
Effect of 50% ethanol extract from fermented *Curcuma longa* L. (FCE50) on mRNA expression of PPAR-γ and C/EBPα in white adipose tissue of high-fat diet-induced obese rats. The normal diet group (ND) comprised rats fed the AIN76 diet; the high-fat diet-induced obese group (HFD) comprised rats fed a 60% high-fat diet; the *Garcinia cambogia* treated group (positive control) (GC) comprised rats fed a 60% high-fat diet with *Garcinia cambogia* 500 g/kg b.w./day; the FCE50-treated group comprised rats fed a 60% high-fat diet with FCE50 500 g/kg b.w./day. All data are expressed as mean±standard deviation (*n*=6). Different letters show a significant difference at *p*<0.05 as determined by Duncan's multiple range test.

There were significant increases in the mRNA expression of PPAR-γ and C/EBPα in the white adipose tissue of the HFD group (PPAR-γ: 1.01±0.09, C/EBPα: 1.03±0.16) compared with the ND group (PPAR-γ: 0.43±0.06, C/EBPα: 0.44±0.18). In the GC (PPAR-γ: 0.23±0.11, C/EBPα: 0.68±0.09) and FCE50 (PPAR-γ: 0.29±0.13, C/EBPα: 0.63±0.13) groups, the expression of PPAR-γ and C/EBPα mRNAs in the white adipose tissue was significantly decreased compared with the HFD group (*p*<0.05; Fig. 1).

### Effect of FCE50 on triglyceride accumulation in high-fat diet-induced obese rats

**Fig. 2:**
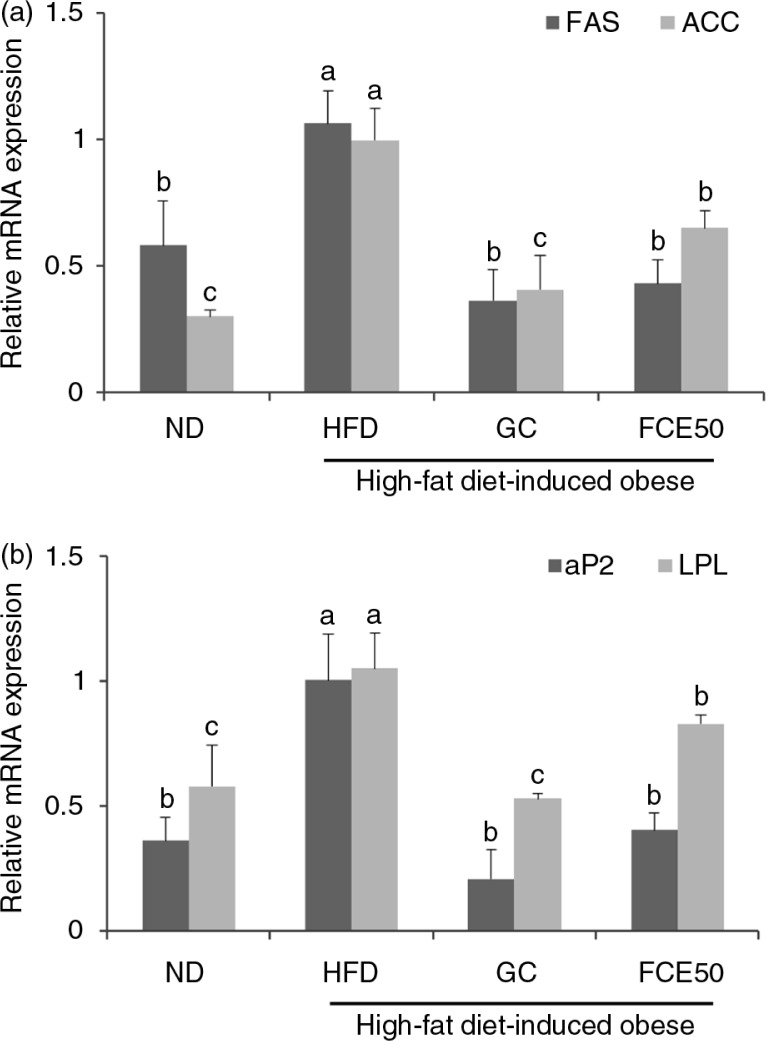
Effect of 50% ethanol extract from fermented *Curcuma longa* L. (FCE50) on mRNA expression of (a) FAS and ACC and (b) aP2 and LPL in white adipose tissue of high-fat diet-induced obese rats. The normal diet group (ND) comprised rats fed the AIN76 diet; the high-fat diet-induced obese group (HFD) comprised rats fed a 60% high-fat diet; the *Garcinia cambogia* treated group (positive control) (GC) comprised rats fed a 60% high-fat diet with *Garcinia cambogia* 500 g/kg b.w./day; the FCE50-treated group comprised rats fed a 60% high-fat diet with FCE50 500 g/kg b.w./day. All data are expressed as mean±standard deviation (*n*=6). Different letters show a significant difference at *p*<0.05 as determined by Duncan's multiple range test.

The mRNA expression of the lipogenic enzymes, FAS and ACC, in the white adipose tissue of the HFD group (FAS: 1.06±0.13, ACC: 1.00±0.13) increased significantly when compared with the ND group (FAS: 0.58±0.17, ACC: 0.30±0.02). There were significant decreases in the mRNA expression of lipogenic enzymes in the white adipose tissue of the GC (FAS: 0.36±0.12, ACC: 0.41±0.13) and FCE50 (FAS: 0.43±0.09, ACC: 0.65±0.07) groups compared with the HFD group (*p*<0.05; Fig. 2a).

The mRNA expression of adipocyte protein 2 (aP2) and LPL in the white adipose tissue of the HFD group (aP2: 1.00± 0.19, LPL: 1.05±0.14) was significantly higher than that of the ND group (aP2: 0.36±0.10, LPL: 0.58±0.17). The mRNA expression of aP2 and LPL in the white adipose tissues of the GC (aP2: 0.21± 0.12, LPL: 0.53±0.02) and FCE50 (aP2: 0.40±0.07, LPL: 0.83± 0.04) groups decreased significantly compared with that of the HFD group (*p*<0.05; Fig. 2b).

### Effect of FCE50 on triglyceride hydrolysis in high-fat diet-induced obese rats

**Fig. 3:**
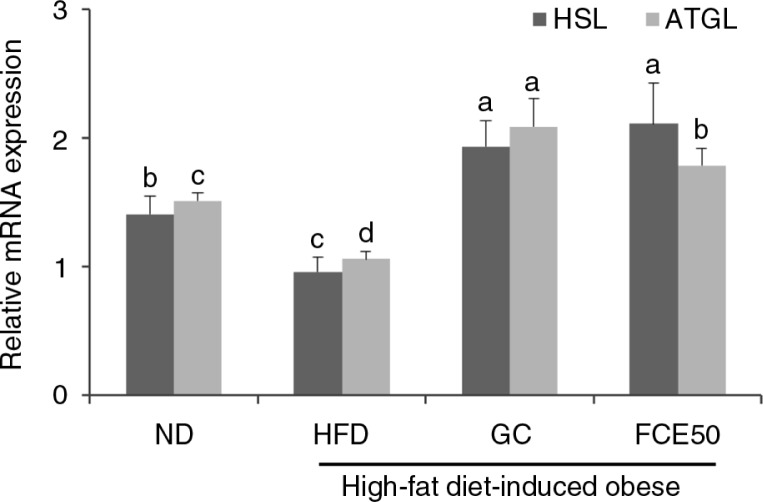
Effect of 50% ethanol extract from fermented *Curcuma longa* L. (FCE50) on mRNA expression of HSL and ATGL in white adipose tissue of high-fat diet-induced obese rats. The normal diet group (ND) comprised rats fed the AIN76 diet; the high-fat diet-induced obese group (HFD) comprised rats fed a 60% high-fat diet; the *Garcinia cambogia* treated group (positive control) (GC) comprised rats fed a 60% high-fat diet with *Garcinia cambogia* 500 g/kg b.w./day; the FCE50-treated group comprised rats fed a 60% high-fat diet with FCE50 500 g/kg b.w./day. All data are expressed as mean±standard deviation (*n*=6). Different letters show a significant difference at *p*<0.05 as determined by Duncan's multiple range test.

The mRNA expression of enzymes for the lipolysis of triglycerides (HSL and ATGL) in the white adipose tissue of the HFD group (HSL: 0.96±0.11, ATGL: 1.05±0.06) decreased significantly compared with that of the ND group (HSL: 1.40±0.14, ATGL: 1.51±0.06). However, the GC group (HSL: 1.93±0.20, ATGL: 2.09±0.22) and the FCE50 group (HSL: 2.11±0.32, ATGL: 1.79±0.13) demonstrated significantly increased mRNA expression of HSL and ATGL in the white adipose tissue compared with the ND group as well as the HFD group (*p*<0.05; Fig. 3).

### Effect of FCE50 on β-oxidation of fatty acids in high-fat diet-induced obese rats

**Fig. 4:**
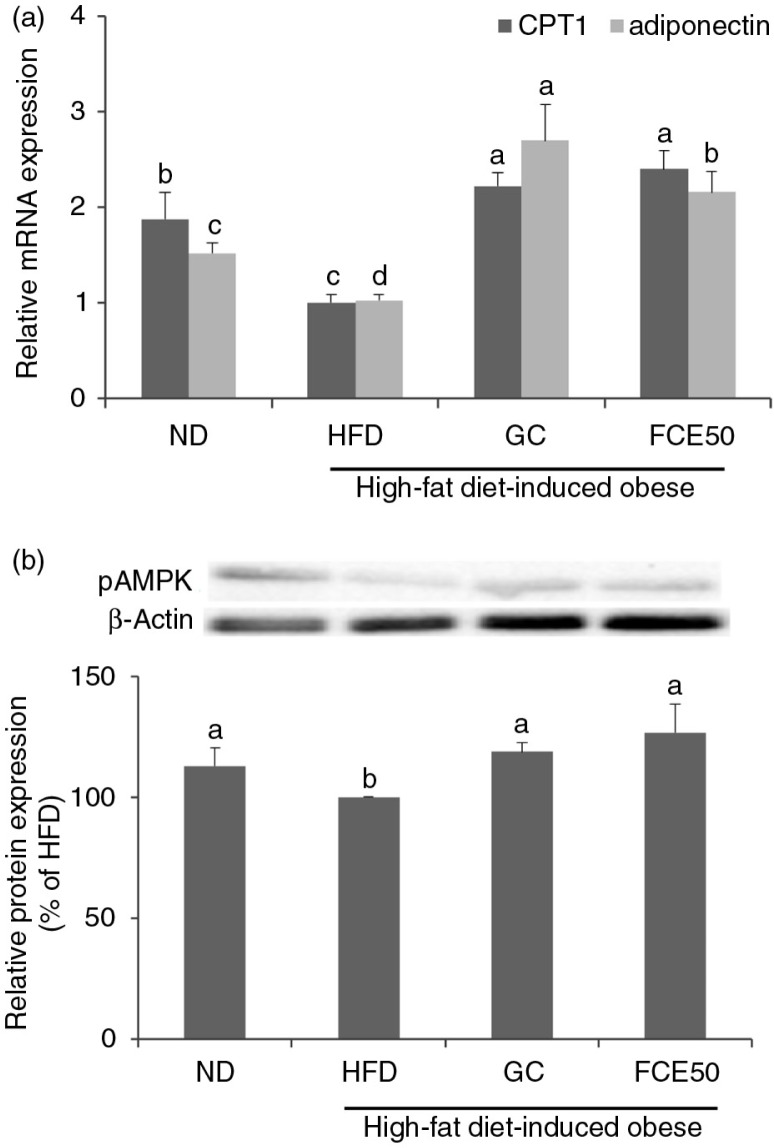
Effect of 50% ethanol extract from fermented *Curcuma longa* L. (FCE50) on (a) mRNA expression of CPT1 and adiponectin and (b) phosphorylation of AMPK in white adipose tissue of high-fat diet-induced obese rats. The normal diet group (ND) comprised rats fed the AIN76 diet; the high-fat diet-induced obese group (HFD) comprised rats fed a 60% high-fat diet; the *Garcinia cambogia* treated group (positive control) (GC) comprised rats fed a 60% high-fat diet with *Garcinia cambogia* 500 g/kg b.w./day; the FCE50-treated group comprised rats fed a 60% high-fat diet with FCE50 500 g/kg b.w./day. All data are expressed as mean±standard deviation (*n*=6). Different letters show a significant difference at *p*<0.05 as determined by Duncan's multiple range test.

There were significant decreases in the mRNA expression of CPT1 and adiponectin as well as in the protein expression of AMPK phosphorylation in the white adipose tissue of the HFD group (CPT1: 1.00±0.09, adiponectin: 1.02±0.06, AMPK phosphorylation: 100%) compared with the ND group (CPT1: 1.88±0.28, adiponectin: 1.52±0.11, AMPK phosphorylation: 112.93±7.39%). The mRNA expression of CPT1 and adiponectin and the protein expression of AMPK phosphorylation in the white adipose tissue of the GC (CPT1: 2.22±0.14, adiponectin: 2.70±0.37, AMPK phosphorylation: 118.78±3.71%) and FCE50 (CPT1: 2.40±0.19, adiponectin: 2.15±0.22, AMPK phosphorylation: 126.75±11.70%) groups significantly increased compared with the HFD group (*p*<0.05; Fig. 4).

## Discussion

Chronic consumption of a high-fat diet has been shown to produce obesity through increases in adipocyte number and size resulting from the high caloric density (17). A high-fat diet-induced obese animal is pathophysiologically very similar to an obese human. Therefore, it is an appropriate animal model for the evaluation of anti-obesity interventions in an *in vivo* experimental test (18). In the present study, we used a high-fat diet-fed SD rat model to investigate the effect of FCE50 on obesity. SD rats have been used to study obesity as they readily gain weight on high-fat diets and are useful animals for studying the pathogenesis of high-fat diet-induced obesity (19). The present study demonstrated significant increases in the weight gain, FER, and white adipose tissue weight in the 60% high-fat diet-fed rats compared with the normal diet-fed rats (Table 1). In addition, we found that the 60% high-fat diet induced dyslipidemia, which is the elevation of triglycerides, total cholesterol, and LDL/VLDL cholesterol in addition to a low HDL level in the blood (Table 2). Ghibaudi et al. (19) also reported that administration of an increasing amount of dietary fat in rats enhanced weight gain, fat mass, plasma cholesterol, triglycerides, and free fatty acid levels. Chronic consumption of a high-fat diet is regarded as a direct cause in the development of dyslipidemia, which can contribute to the development of abnormal lipid metabolism, atherosclerosis, and obesity (20).

On the other hand, we found a marked decrease in the weight gain, FER, and white adipose tissue weight as well as improvements in the development of dyslipidemia in the 60% high-fat diet-fed rat treated with FCE50 (Tables 1 and 2). The reduction of weight gain and dyslipidemia induced by FCE50 administration can be correlated with the inhibition of abnormal fat accumulation in adipose tissue and the development of obesity-related diseases.

Adipose tissue has important functions such as helping to reduce heat loss, providing mechanical protection, and energy storage. Although adipose tissue is vitally important for various normal processes, excess adipose tissue can have many negative implications for human disease states with the development of obesity (21). The mechanism of adipose tissue expansion has been studied for decades to understand how it contributes to the metabolic dysregulation associated with obesity. The expansion of adipose tissue begins when differentiated adipocytes rapidly respond to nutrient excess (22).

When pre-adipocytes differentiate into mature adipocytes, the mature cells can act as lipid-synthesizing and lipid-storing adipocytes (4). The differentiation of pre-adipocytes is regulated by several transcription factors such as PPAR-γ and C/EBPα that play a central role in the control of adipogenesis (6). Kadowaki et al. (23) reported that PPAR-γ mediates high-fat-diet-induced adipocyte differentiation and adipocyte hypertrophy to generate large adipocytes and insulin resistance. In addition, the report from Rosen et al. (24) demonstrated that PPAR-γ and C/EBPα participated in a single pathway during the differentiation of pre-adipocytes, with PPAR-γ being the proximal effector of adipogenesis whereas C/EBPα has no ability to promote differentiation in the absence of PPAR-γ. We found in the present study that the 60% high-fat diet-fed rats exhibited a significant increase in the mRNA expression of PPAR-γ and C/EBPα in white adipose tissue compared with the normal diet-fed rats (Fig. 1). Moreover, the mRNA expression of FAS, ACC, aP2, and LPL in white adipose tissue was increased by chronic high-fat diet intake (Fig. 2). PPAR-γ and C/EBPα regulate the transcription factors involved in adipose tissue adipogenesis such as FAS, ACC, aP2, and LPL. FAS and ACC are enzymes for the synthesis of fatty acids from acetyl-CoA and malonyl-CoA, which is an important step of the lipogenesis process (25). When LPL hydrolyzes triglycerides in lipoproteins in the blood to promote cellular uptake of fatty acids and triglyceride, aP2 acts as the carrier protein for fatty acids in adipocytes. Thus, LPL and aP2 are genes closely associated with fatty acid uptake and triglyceride accumulation (26).

Interestingly, this present study found that FCE50 administration induced a statistically significant decrease in the mRNA expressions of FAS, ACC, aP2, and LPL as well as PPAR-γ and C/EBPα (Figs. 1 and 2). Some studies have reported that *C. longa* L. and its biologically active compounds such as curcumin have an effect on the inhibition of FAS, differentiation and triglyceride accumulation in adipocytes (13, 27, 28). Ejaz et al. (27) found that dietary curcumin caused lower adiposity and weight gain coupled with the suppression of PPAR-γ and C/EBPα expression in high-fat diet-fed mice. A report by Zhao et al. (28) revealed that curcumin works as a FAS inhibitor, suppressing the differentiation of pre-adipocytes and lipid accumulation in adipocytes. According to these reports and our present results, FCE50 administration reduces weight gain and fat accumulation by the suppression of adipocyte differentiation and lipogenesis due to the effect of curcumin.

In addition, we found that FCE50 administration leads to the activation of lipolysis in the adipose tissue of high-fat diet-fed rats (Figs. 3 and 4). White adipose tissue is metabolic flexible tissue, which is important for energy storage or energy source release in the form of anabolism or catabolism of triglycerides (29). Catabolism of triglycerides, known as lipolysis, is regulated in consecutive steps that involve at least three different enzymes such as ATGL, HSL, and monoacylglycerol lipase (MGL), resulting in the release of glycerol and fatty acids (9). Catabolism of fatty acids, known as β-oxidation, generates acetyl-CoA, which enters the TCA cycle and produces ATP (30). An imbalance between fatty acid uptake, synthesis, and β-oxidation can be associated with triglyceride accumulation in adipocyte (30). It has been reported that the phosphorylation of AMPK leads to β-oxidation by inactivating ACC and up-regulating CPT1 expression. AMPK is a multisubunit enzyme recognized as a major regulator of lipid biosynthetic pathways due to its role in the phosphorylation and inactivation of key enzymes (9, 10, 31). Herms et al. (31) recently reported that lipid droplets that efficiently supply fatty acids were induced by the activation of the energy sensor AMPK for mitochondrial β-oxidation during nutrient starvation.

**Fig. 5:**
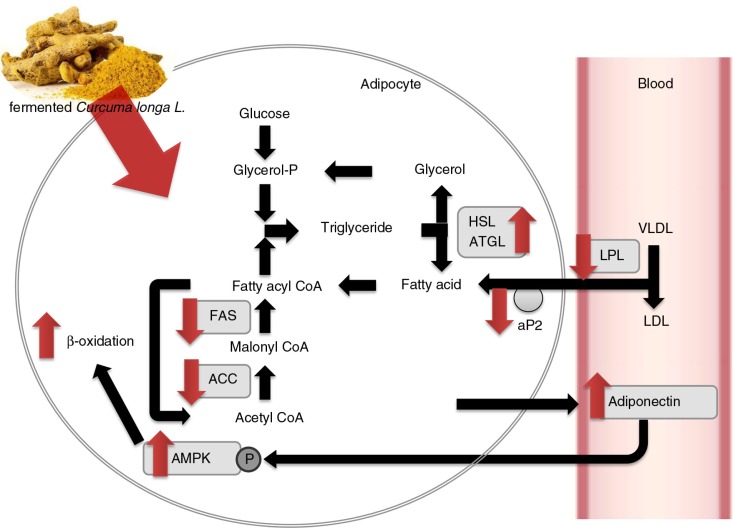
Effect of 50% ethanol extract from fermented *Curcuma longa* L. (FCE50) on lipid metabolism. FCE50 suppressed lipogenesis with a decrease in the expressions of fatty acid synthase (FAS), acetyl-CoA carboxylase (ACC), adipocyte protein 2 (aP2), and lipoprotein lipase (LPL), and increased lipolysis and β-oxidation by up-regulating the expression of lipases such as adipose triglyceride lipase (ATGL), hormone-sensitive lipase (HSL), adiponectin, and AMP-activated protein kinase (AMPK) phosphorylation.

Adiponectin, a novel adipocyte-specific protein, has been shown to increase β-oxidation via the phosphorylation of AMPK and inactivation of ACC (32, 33). It has been reported that adiponectin levels are lower in obese subjects than in lean subjects (34). In the present study, we found that the expression of adiponectin was suppressed in the white adipose tissue of the high-fat diet-fed rats. In addition, mRNA expression of CPT1 and the protein expression of AMPK phosphorylation were decreased in the white adipose tissue of the HFD group compared with the ND group. However, we found that mRNA expression of CPT1 and adiponectin as well as the protein expression of AMPK phosphorylation were significantly higher in the FCE50 group (Fig. 5). These results indicate that FCE50 administration inhibits lipogenesis and enhanced lipolysis through adiponectin and pAMPK expression in the white adipose tissue.

In conclusion, we found that FCE50 administration suppressed b.w. gain, white adipose tissue weight, serum TG, and cholesterol in high-fat diet-induced obese rats. These results can be associated with the suppression of adipocyte differentiation and lipogenesis with the decrease in the mRNA expressions of FAS, ACC, aP2, and LPL induced by FCE50 administration. In addition, FCE50 increased lipolysis and β-oxidation by up-regulating the expression of lipases such as ATGL and HSL, adiponectin, and AMPK phosphorylation. The present study demonstrated that FCE50 could be a good option for preventing adipogenesis and activating lipolysis.

## References

[1] Must A, Spadano J, Coakley EH, Field AE, Colditz G, Dietz WH (1999). The disease burden associated with overweight and obesity. JAMA.

[2] Rose DP, Gracheck PJ, Vona-Davis L (2015). The interactions of obesity, inflammation and insulin resistance in breast cancer. Cancers (Basel).

[3] Frühbeck G, Gómez-Ambrosi J, Muruzábal FJ, Burrell MA (2001). The adipocyte: a model for integration of endocrine and metabolic signaling in energy metabolism regulation. Am J Physiol Endocrinol Metab.

[4] Ntambi JM, Young-Cheul K (2000). Adipocyte differentiation and gene expression. J Nutr.

[5] Darlington GJ, Ross SE, MacDougald OA (1998). The role of C/EBP genes in adipocyte differentiation. J Biol Chem.

[6] Morrison RF, Farmer SR (2000). Hormonal signaling and transcriptional control of adipocyte differentiation. J Nutr.

[7] Hietanen E, Greenwood MR (1977). A comparison of lipoprotein lipase activity and adipocyte differentiation in growing male rats. J Lipid Res.

[8] Duncan RE, Ahmadian M, Jaworski K, Sarkadi-Nagy E, Sul HS (2007). Regulation of lipolysis in adipocytes. Annu Rev Nutr.

[9] Gaidhu MP, Anthony NM, Patel P, Hawke TJ, Ceddia RB (2010). Dysregulation of lipolysis and lipid metabolism in visceral and subcutaneous adipocytes by high-fat diet: role of ATGL, HSL, and AMPK. Am J Physiol Cell Physiol.

[10] Ceddia RB (2013). The role of AMP-activated protein kinase in regulating white adipose tissue metabolism. Mol Cell Endocrinol.

[11] Kohjima M, Higuchi N, Kato M, Kotoh K, Yoshimoto T, Fujino T (2008). SREBP-1c, regulated by the insulin and AMPK signaling pathways, plays a role in nonalcoholic fatty liver disease. Int J Mol Med.

[12] Yun JW (2010). Possible anti-obesity therapeutics from nature – a review. Phytochemistry.

[13] Witkin JM, Li X (2013). Curcumin, an active constiuent of the ancient medicinal herb Curcuma longa L.: some uses and the establishment and biological basis of medical efficacy. CNS Neurol Disord Drug Targets.

[14] Araújo CC, Leon LL (2001). Biological activities of *Curcuma longa* L. Mem Inst Oswaldo Cruz.

[15] Kim Y, You Y, Yoon HG, Lee YH, Kim K, Lee J (2014). Hepatoprotective effects of fermented *Curcuma longa* L. on carbon tetrachloride-induced oxidative stress in rats. Food Chem.

[16] Chuah LO, Yeap SK, Ho WY, Beh BK, Alitheen NB (2012). *In vitro* and *in vivo* toxicity of garcinia or hydroxycitric Acid: a review. Evid Based Complement Alternat Med.

[17] Bray GA, Popkin BM (1998). Dietary fat intake does affect obesity!. Am J Clin Nutr.

[18] Varga O, Harangi M, Olsson IA, Hansen AK (2010). Contribution of animal models to the understanding of the metabolic syndrome: a systematic overview. Obes Rev.

[19] Ghibaudi L, Cook J, Farley C, van Heek M, Hwa JJ (2002). Fat intake affects adiposity, comorbidity factors, and energy metabolism of Sprague-Dawley rats. Obes Res.

[20] Yatera Y, Shibata K, Furuno Y, Sabanai K, Morisada N, Nakata S (2010). Severe dyslipidaemia, atherosclerosis, and sudden cardiac death in mice lacking all NO synthases fed a high-fat diet. Cardiovasc Res.

[21] Rosen ED, Spiegelman BM (2006). Adipocytes as regulators of energy balance and glucose homeostasis. Nature.

[22] Rutkowski JM, Stern JH, Scherer P (2015). The cell biology of fat expansion. J Cell Biol.

[23] Kadowaki T, Hara K, Kubota N, Tobe K, Terauchi Y, Yamauchi T (2002). The role of PPARgamma in high-fat diet-induced obesity and insulin resistance. J Diabetes Complications.

[24] Rosen ED, Hsu CH, Wang X, Sakai S, Freeman MW, Gonzalez FJ (2002). C/EBPalpha induces adipogenesis through PPARgamma: a unified pathway. Genes Dev.

[25] Schadinger SE, Bucher NL, Schreiber BM, Farmer SR (2005). PPARgamma2 regulates lipogenesis and lipid accumulation in steatotic hepatocytes. Am J Physiol Endocrinol Metab.

[26] Farmer SR (2006). Transcriptional control of adipocyte formation. Cell Metab.

[27] Ejaz A, Wu D, Kwan P, Meydani M (2009). Curcumin inhibits adipogenesis in 3T3-L1 adipocytes and angiogenesis and obesity in C57/BL mice. J Nutr.

[28] Zhao J, Sun XB, Ye F, Tian WX (2011). Suppression of fatty acid synthase, differentiation and lipid accumulation in adipocytes by curcumin. Mol Cell Biochem.

[29] Redinger RN (2009). Fat storage and the biology of energy expenditure. Transl Res.

[30] Abu-Elheiga L, Matzuk MM, Abo-Hashema KA, Wakil SJ (2001). Continuous fatty acid oxidation and reduced fat storage in mice lacking acetyl-CoA carboxylase 2. Science.

[31] Herms A, Bosch M, Reddy BJ, Schieber NL, Fajardo A, Rupérez C (2015). AMPK activation promotes lipid droplet dispersion on detyrosinated microtubules to increase mitochondrial fatty acid oxidation. Nat Commun.

[32] Yoon MJ, Lee GY, Chung JJ, Ahn YH, Hong SH, Kim JB (2006). Adiponectin increases fatty acid oxidation in skeletal muscle cells by sequential activation of AMP-activated protein kinase, p38 mitogen-activated protein kinase, and peroxisome proliferator-activated receptor alpha. Diabetes.

[33] Lago F, Gómez R, Gómez-Reino JJ, Dieguez C, Gualillo O (2009). Adipokines as novel modulators of lipid metabolism. Trends Biochem Sci.

[34] Yu JG, Javorschi S, Hevener AL, Kruszynska YT, Norman RA, Sinha M (2002). The effect of thiazolidinediones on plasma adiponectin levels in normal, obese, and type 2 diabetic subjects. Diabetes.

